# Factors associated with early puncture-site wound events after percutaneous vertebroplasty for osteoporotic vertebral compression fractures: a retrospective cohort study

**DOI:** 10.3389/fmed.2026.1988843

**Published:** 2026-10-21

**Authors:** Xiangle He, Changzhi Huang, Bingqian Zheng, Xianliang Wu, Chengguo Huang, Shuqiang Lu, Yufeng Huang, Jinghan Chen, Jianjun Huang, Bei Li

**Affiliations:** 1 Ningde Clinical Medical College of Fujian Medical University, Ningde, China; 2 Department of Orthopedics, Ningde Municipal Hospital, Ningde, China; 3 Department of Rehabilitation Medicine, Ningde Municipal Hospital, Ningde, China; 4 Department of Orthopedics, Pingnan County Hospital, Ningde, China

**Keywords:** osteoporotic vertebral compression fractures, percutaneous vertebroplasty, puncture-site wound events, risk factors, wound closure

## Abstract

**Objective:**

Early puncture-site wound events after percutaneous vertebroplasty (PVP) for osteoporotic vertebral compression fractures (OVCFs) often receive less attention than major complications, but they may influence early wound monitoring and postoperative risk assessment. This study aimed to identify factors associated with early puncture-site wound events after elective PVP.

**Methods:**

This single-center retrospective cohort study included consecutive patients who underwent elective PVP for OVCFs between January 2022 and June 2026. The primary outcome was any puncture-site wound event within 2 weeks after surgery, defined as erythema or swelling, bleeding or exudate, subcutaneous hematoma, or infection. Prespecified demographic, clinical, laboratory, and procedure-related candidate factors were first evaluated in univariable screening analyses. Modified Poisson regression with robust standard errors was then used to estimate adjusted risk ratios (RRs) and 95% confidence intervals (CIs) for variables meeting the screening criterion or selected *a priori* for clinical relevance.

**Results:**

Among 1,307 eligible patients, 136 (10.4%) had recorded early puncture-site wound events. Suture closure (RR: 2.24; 95% CI: 1.59–3.16; *P* < 0.001), diabetes (RR: 1.57; 95% CI: 1.11–2.21; *P* = 0.010), elevated international normalized ratio (RR: 1.66; 95% CI: 1.08–2.57; *P* = 0.022), and multilevel treatment (RR: 1.73; 95% CI: 1.03–2.89; *P* = 0.038) were associated with a higher adjusted risk of the composite outcome. Age, sex, and body mass index were not independently associated with the composite outcome in the primary model. In the crude comparison by closure method, any event occurred in 53 of 774 patients (6.8%) with nonsuture closure and in 83 of 533 patients (15.6%) with suture closure (crude RR: 2.27; 95% CI: 1.64–3.15; *P* < 0.001).

**Conclusion:**

Early wound status after elective PVP for OVCFs was associated with metabolic and coagulation status, treatment extent, and local puncture-site closure method. These findings should be interpreted within the limits of a retrospective observational study.

## 1 Introduction

Osteoporotic vertebral compression fractures (OVCFs) are among the most common fragility fractures in older adults and may lead to persistent back pain, restricted spinal mobility, prolonged bed rest, and impaired quality of life (1, 2). For patients with inadequate pain control or limited functional recovery after conservative treatment, percutaneous vertebroplasty (PVP) has become a widely used minimally invasive option because it causes limited tissue trauma, requires a relatively short operating time, rapidly stabilizes the fractured vertebra, and facilitates early mobilization (3–5). Perioperative management in older patients emphasizes rapid recovery, shorter hospitalization, and lower postoperative management burden. Therefore, postoperative assessment after PVP should not focus only on pain relief and radiographic complications, but should also consider puncture-site healing, a local issue directly relevant to short-term wound management and postoperative follow-up.

The puncture site after PVP is usually small, and most patients heal uneventfully. Nevertheless, early wound events remain clinically relevant in real-world practice. Surgical site infection (SSI) is an important safety outcome, but its incidence after vertebral augmentation is low (6, 7). By contrast, erythema or swelling, exudate, and hematoma are more commonly encountered during clinical observation. Even when these events do not lead to severe consequences, they may prompt additional monitoring, wound care, post-discharge consultation, uncertainty during follow-up, and patient concern about wound healing (8–10). Such events may not alter the overall therapeutic effect of PVP, but they can affect the smoothness of postoperative management. In the predominantly older osteoporotic population undergoing PVP, postoperative wound recovery may also be influenced by skin condition, nutritional status, comorbidities, and perioperative coagulation status (9, 11, 12). Therefore, using early puncture-site wound events as an outcome may better capture short-term local wound status after PVP than focusing on infection alone.

Previous PVP studies have mainly focused on pain relief, vertebral height restoration, cement leakage, neurologic injury, refracture, and overall perioperative safety (5, 13–17). In contrast, early postoperative puncture-site wound events have received limited attention. Existing reports often describe puncture-site management only briefly as part of the procedural workflow, and systematic analyses of the incidence and associated factors of early puncture-site wound events are lacking. In real-world practice, baseline patient status, coagulation reserve, treatment extent, and local puncture-site closure method may all be related to short-term postoperative wound appearance, but the associations between these factors and early puncture-site wound events remain insufficiently supported by data. Identifying these factors may help move postoperative wound observation from experience-based judgment toward more evidence-informed risk recognition and may provide a clearer basis for wound documentation and follow-up priorities.

Accordingly, this study evaluated the occurrence of puncture-site wound events within 2 weeks after surgery in a cohort of patients with OVCFs who underwent elective PVP. Erythema or swelling, bleeding or exudate, subcutaneous hematoma, and infection were combined into a composite outcome, and clinical and perioperative factors associated with this outcome were analyzed. This study therefore aimed to quantify a 2-week patient-level composite outcome of puncture-site wound events after elective PVP and examine its adjusted associations with patient-related factors and closure method.

## 2 Methods

### 2.1 Study design and patients

This single-center retrospective cohort study included consecutive patients who underwent elective PVP for OVCFs at Ningde Municipal Hospital between January 2022 and June 2026. The exclusion criteria were second surgery for new fracture, uncontrolled coagulation disorders, concomitant open spinal surgery, intraoperative puncture-site incision, malignant tumor or abnormal immune status, and missing key clinical data. This manuscript was prepared in accordance with the Strengthening the Reporting of Observational Studies in Epidemiology (STROBE) statement. The study protocol was reviewed and approved by the hospital ethics committee, with a waiver of written informed consent (approval No. NSYKYLL-2026-110).

### 2.2 Data collection and definitions

Clinical and perioperative data were obtained from the electronic medical record system, operative records, anesthesia records, laboratory reports, and postoperative wound management records. Baseline variables included age, sex, body mass index (BMI), smoking history, alcohol dependence, diabetes, elevated international normalized ratio (INR), white blood cell (WBC) count, platelet (PLT) count, hemoglobin (HB), albumin (ALB), American Society of Anesthesiologists (ASA) class, preoperative antibiotic use, primary surgeon experience >15 years, surgical incision class, unilateral puncture, treated level, and puncture-site closure method. Elevated INR was defined as >1.20 according to the upper limit of the institutional laboratory reference range. Treated level was recorded as a four-category variable: single level at T10 or above, single level at T11-L2, single level at L3 or below, and multilevel treatment. According to operative and wound management records, puncture-site closure was classified as nonsuture closure or suture closure. Diabetes, smoking history, alcohol dependence, immune status abnormality, and uncontrolled coagulation disorder were identified from admission diagnoses, medical history, and perioperative assessment records before the index PVP. Laboratory variables used the most recent preoperative test results. Preoperative antibiotic use referred to antibiotic treatment documented before the index procedure rather than standardized perioperative prophylaxis. Surgical incision class was assessed before puncture or incision according to anticipated contamination risk.

Puncture-site management and follow-up were further reviewed from operative records and postoperative wound management records. Closure was determined by the operating physician based on intraoperative findings and routine practice, including bleeding, exudation, skin condition, and wound tension. All puncture sites were covered with waterproof dressings after surgery. Sutured sites were typically closed with one 3-0 Vicryl Rapide stitch. Puncture sites were assessed within 2 weeks after PVP by inpatient observation, wound-clinic review, or telephone follow-up. In-person assessments included dressing and wound examination, whereas telephone follow-up assessed patient-reported erythema, swelling, bleeding, exudate, hematoma, infection, and other wound concerns.

### 2.3 Outcome

The primary outcome was any puncture-site wound event within 2 weeks after surgery. This patient-level composite outcome was defined as the occurrence of at least one of the following events: puncture-site erythema or swelling, bleeding or exudate, subcutaneous hematoma, or infection. The composite outcome was counted once per patient, regardless of whether one or more component events occurred. Each component wound event was summarized separately according to closure method. Puncture-site wound events were identified from inpatient records, postoperative dressing-change records, wound-clinic records, and documented telephone follow-up information within 2 weeks after PVP. Events were counted only when explicitly documented in these records. Patients without key clinical data or valid wound follow-up information were excluded because of missing key data. Ambiguous or disputed records were reviewed by the study team. Infection required a documented postoperative clinical diagnosis supported by microbiological evidence.

### 2.4 Statistical analysis

Baseline and perioperative characteristics were compared between patients with and without any early puncture-site wound event. Categorical variables are presented as counts and percentages and were compared using the Pearson chi-square test or Fisher exact test, as appropriate. Continuous variables were first assessed for distribution using the Shapiro–Wilk test within each outcome group. When both groups showed normal distributions, continuous variables are presented as mean ± standard deviation and were compared using the Welch *t* test; otherwise, they are presented as median and interquartile range (IQR) and were compared using the Mann–Whitney *U* test. Standardized mean differences (SMDs) were reported to describe between-group imbalance.

Factors associated with the primary outcome were evaluated using multivariable modified Poisson regression with robust standard errors, and effect estimates are presented as adjusted risk ratios (RRs) and 95% confidence intervals (CIs). Before fitting the multivariable model, candidate factors were assessed by univariable screening. Each candidate variable was evaluated separately using univariable modified Poisson regression with robust standard errors, and crude RRs, 95% CIs, and *P* values were reported. Variables that reached the prespecified statistical threshold of *P* < 0.05 in univariable analysis and were clinically relevant were considered for inclusion in the multivariable model. In addition, age, sex, and BMI were included as prespecified demographic covariates because of their clinical relevance, irrespective of their univariable *P* values. The final primary model included suture closure, diabetes, INR > 1.2, age per 10-year increase, female sex, BMI per 1 kg/m^2^ increase, and multilevel treatment. The reference categories for categorical variables were nonsuture closure, no diabetes, INR ≤ 1.2, male sex, and single-level treatment. Because component wound events occurred less frequently than the composite outcome, the associations between closure method and each component event were assessed only in crude comparisons. In these analyses, incidence was reported as counts and percentages, crude RRs and 95% CIs were calculated, and *P* values were reported.

All statistical analyses were performed using R software, version 4.6.0 (R Foundation for Statistical Computing, Vienna, Austria). All tests were two-sided, and *P* < 0.05 was considered statistically significant.

## 3 Results

A total of 1,472 PVP records were screened, and 165 records were excluded according to the inclusion and exclusion criteria. The final analytical cohort included 1,307 patients who underwent elective PVP for OVCFs. Within 2 weeks after surgery, 136 patients (10.4%) had recorded early puncture-site wound events, whereas 1,171 patients (89.6%) had no events (Figure 1).

**Figure 1:**
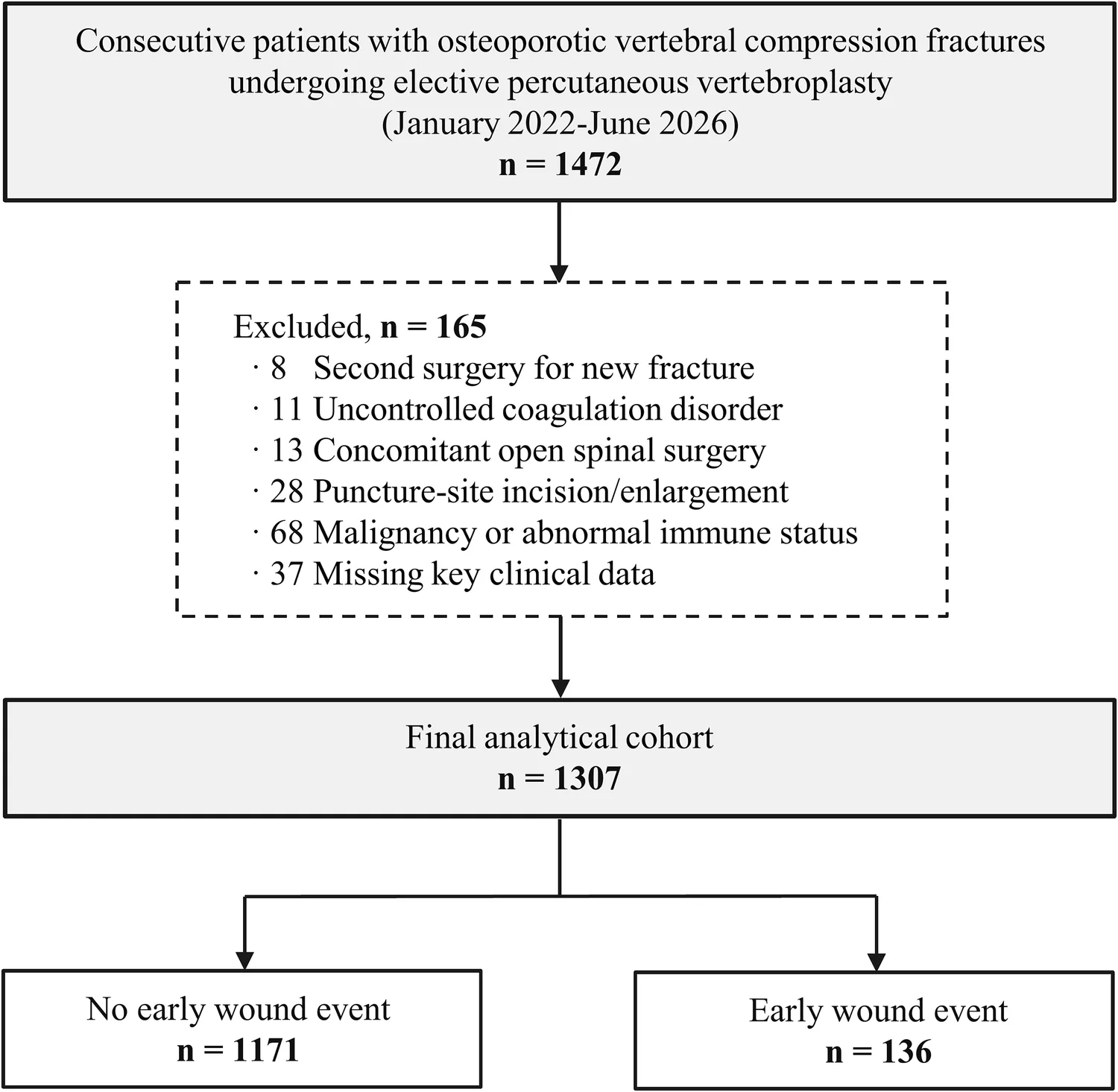
Study flow diagram.

### 3.1 Baseline and perioperative characteristics

Compared with patients without events, those with events had a higher proportion of diabetes (30.9% vs. 21.7%; *P* = 0.015) and elevated INR (16.2% vs. 10.4%; *P* = 0.021). Age, sex, BMI, smoking history, alcohol dependence, WBC count, PLT count, HB, ALB, and ASA class all had *P* values >0.05 (Table 1).

**Table 1:** Baseline characteristics.

Variable	No early wound event (*n* = 1,171)	Early wound event (*n* = 136)	*P* value	SMD
Age, years	74.6 ± 8.6	75.2 ± 7.9	0.381	0.077
Sex			0.701	0.035
Female	904 (77.2%)	103 (75.7%)		
Male	267 (22.8%)	33 (24.3%)		
BMI, kg/m^2^	22.2 ± 2.5	22.6 ± 2.9	0.119	0.151
Smoking history	77 (6.6%)	7 (5.1%)	0.520	0.061
Alcohol dependence	44 (3.8%)	6 (4.4%)	0.707	0.033
Diabetes	254 (21.7%)	42 (30.9%)	0.015	0.209
INR > 1.2	122 (10.4%)	22 (16.2%)	0.021	0.242
WBC, 10^9^/L	6.87 (5.46–8.73)	7.41 (5.70–8.91)	0.106	0.122
PLT, 10^9^/L	222.0 (183.0–266.0)	209.5 (169.5–261.5)	0.080	0.156
HB, g/L	123.1 ± 16.4	120.9 ± 16.1	0.152	0.130
ALB, g/L	40.1 ± 3.6	39.4 ± 4.4	0.097	0.163
ASA class			0.113	0.134
II	1,058 (90.4%)	117 (86.0%)		
III	113 (9.6%)	19 (14.0%)		

SMD, standardized mean difference; BMI, body mass index; INR, international normalized ratio; WBC, white blood cell count; PLT, platelet count; HB, hemoglobin; ALB, albumin; ASA, American Society of Anesthesiologists.

*P* values were calculated using the Pearson chi-square test or Fisher exact test for categorical variables and the Welch *t* test or Mann–Whitney *U* test for continuous variables, as appropriate.

The distribution of treated levels differed between the two groups. Multilevel treatment was recorded in 15 patients with events (11.0%) and 58 patients without events (5.0%) (*P* = 0.009). Suture closure was recorded in 83 patients with events (61.0%) and 450 patients without events (38.4%) (*P* < 0.001). The remaining perioperative characteristics, including preoperative antibiotic use, primary surgeon experience >15 years, surgical incision class, and unilateral puncture, all had *P* values >0.05 (Table 2).

**Table 2:** Perioperative characteristics.

Variable	No early wound event (*n* = 1,171)	Early wound event (*n* = 136)	*P* value	SMD
Preoperative antibiotic use	62 (5.3%)	10 (7.4%)	0.319	0.085
Surgeon experience >15 years	501 (42.8%)	49 (36.0%)	0.131	0.138
Surgical incision class			1.000	0.058
Class I, clean	1,169 (99.8%)	136 (100%)		
Class II, clean-contaminated	2 (0.2%)	0 (0.0%)		
Unilateral puncture	31 (2.6%)	3 (2.2%)	1.000	0.029
Treated level			0.009	0.287
Single level: T10 or above	252 (21.5%)	19 (14.0%)		
Single level: T11-L2	701 (59.9%)	85 (62.5%)		
Single level: L3 or below	160 (13.7%)	17 (12.5%)		
Multilevel	58 (5.0%)	15 (11.0%)		
Suture closure	450 (38.4%)	83 (61.0%)	<0.001	0.452

SMD, standardized mean difference.

*P* values were calculated using the Pearson chi-square test or Fisher exact test, as appropriate.

### 3.2 Univariable screening

As shown in Table 3, univariable modified Poisson regression showed that diabetes, INR > 1.2, multilevel treatment, and suture closure were associated with a higher risk of early puncture-site wound events, with crude RRs of 1.53 (95% CI: 1.09–2.14; *P* = 0.015), 1.56 (95% CI: 1.02–2.38; *P* = 0.039), 2.10 (95% CI: 1.29–3.39; *P* = 0.003), and 2.27 (95% CI: 1.64–3.15; *P* < 0.001), respectively. Age, female sex, and BMI were used as prespecified demographic variables for subsequent adjustment. Because the primary model focused on baseline systemic status, coagulation reserve, treatment extent, and local puncture-site management, the subsequent multivariable model included age, sex, BMI, diabetes, INR > 1.2, multilevel treatment, and suture closure.

**Table 3:** Univariable screening of candidate factors for early puncture-site wound events.

Variable	Crude RR (95% CI)	*P* value	Multivariable model handling
Age, per 10-year increase	1.08 (0.91–1.29)	0.376	Prespecified demographic covariate
Female	0.93 (0.64–1.35)	0.700	Prespecified demographic covariate
BMI, per 1 kg/m^2^ increase	1.06 (0.99–1.14)	0.113	Prespecified demographic covariate
Smoking history	0.79 (0.38–1.64)	0.526	
Alcohol dependence	1.16 (0.54–2.50)	0.704	
Diabetes	1.53 (1.09–2.14)	0.015	*P* < 0.05
INR > 1.2	1.56 (1.02–2.38)	0.039	*P* < 0.05
WBC, per 1 × 10^9^/L increase	1.04 (0.98–1.10)	0.152	
PLT, per 10 × 10^9^/L increase	0.98 (0.95–1.00)	0.096	
HB, per 10 g/L increase	0.93 (0.85–1.02)	0.139	
ALB, per 1 g/L increase	0.96 (0.92–1.01)	0.081	
ASA III vs. II	1.45 (0.92–2.27)	0.109	
Preoperative antibiotic use	1.36 (0.75–2.48)	0.312	
Surgeon experience >15 years	0.78 (0.56–1.08)	0.133	
Class I vs. Class II incision	Not estimable	1.000^†^	
Unilateral puncture	0.84 (0.28–2.52)	0.762	
Multilevel vs. single level	2.10 (1.29–3.39)	0.003	*P* < 0.05
Suture closure	2.27 (1.64–3.15)	<0.001	*P* < 0.05

RR, risk ratio; CI, confidence interval; BMI, body mass index; INR, international normalized ratio; WBC, white blood cell count; PLT, platelet count; HB, hemoglobin; ALB, albumin; ASA, American Society of Anesthesiologists.

Crude RRs, 95% CIs, and *P* values were obtained from univariable modified Poisson regression with robust standard errors unless otherwise indicated.

† *P* value was calculated using the Fisher exact test; the RR was not estimable because one group had zero events.

### 3.3 Multivariable analysis

As shown in Table 4 and Figure 2, in the multivariable modified Poisson regression model with robust standard errors, the adjusted RR for suture closure was 2.24 (95% CI: 1.59–3.16; *P* < 0.001). Diabetes, elevated INR, and multilevel treatment were also associated with higher event risk, with adjusted RRs of 1.57 (95% CI: 1.11–2.21; *P* = 0.010), 1.66 (95% CI: 1.08–2.57; *P* = 0.022), and 1.73 (95% CI: 1.03–2.89; *P* = 0.038), respectively. The adjusted RRs were 1.15 (95% CI: 0.94–1.41; *P* = 0.186) for each 10-year increase in age, 0.96 (95% CI: 0.66–1.39; *P* = 0.829) for female sex, and 1.07 (95% CI: 0.99–1.17; *P* = 0.091) for each 1 kg/m^2^ increase in BMI.

**Table 4:** Multivariable analysis of factors associated with early puncture-site wound events.

Variable	Adjusted RR (95% CI)	*P* value
Suture closure	2.24 (1.59–3.16)	<0.001
Diabetes	1.57 (1.11–2.21)	0.010
INR > 1.2	1.66 (1.08–2.57)	0.022
Age, per 10-year increase	1.15 (0.94–1.41)	0.186
Female	0.96 (0.66–1.39)	0.829
BMI, per 1 kg/m^2^ increase	1.07 (0.99–1.17)	0.091
Multilevel treatment	1.73 (1.03–2.89)	0.038

RR, risk ratio; CI, confidence interval; INR, international normalized ratio; BMI, body mass index.

Adjusted RRs, 95% CIs, and *P* values were obtained from multivariable modified Poisson regression with robust standard errors.

**Figure 2:**
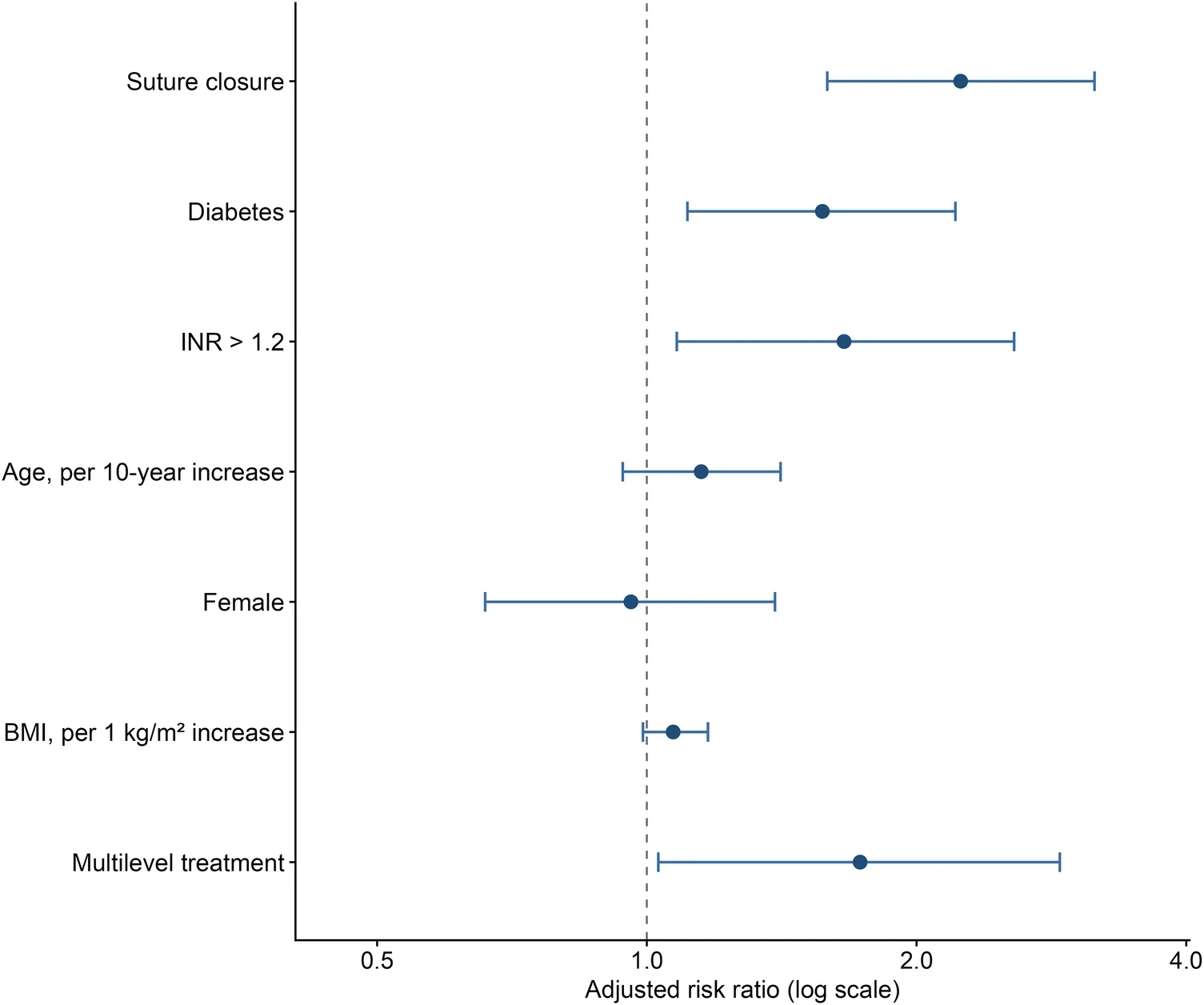
Adjusted risk ratios for early puncture-site wound events. INR, international normalized ratio; BMI, body mass index.

### 3.4 Event types by closure method

As shown in Table 5 and Figure 3, when patients were compared by closure method, any event occurred in 53 of 774 patients (6.8%) with nonsuture closure and in 83 of 533 patients (15.6%) with suture closure [crude RR: 2.27 (95% CI: 1.64–3.15); *P* < 0.001]. Erythema or swelling occurred in 32 patients (4.1%) and 58 patients (10.9%), respectively [crude RR: 2.63 (95% CI: 1.73–3.99); *P* < 0.001]. Bleeding or exudate occurred in 21 patients (2.7%) and 31 patients (5.8%), respectively [crude RR: 2.14 (95% CI: 1.25–3.69); *P* = 0.005]. Subcutaneous hematoma occurred in 2 patients (0.3%) with nonsuture closure and 3 patients (0.6%) with suture closure [crude RR: 2.18 (95% CI: 0.37–12.99); *P* = 0.403]. Infection occurred in 1 patient (0.1%) with nonsuture closure and in 0 patients with suture closure (*P* = 1.000).

**Table 5:** Early puncture-site wound events by closure method.

Early wound event	Nonsuture closure (*n* = 774)	Suture closure (*n* = 533)	Crude RR (95% CI)	*P* value
Any event	53 (6.8%)	83 (15.6%)	2.27 (1.64–3.15)	<0.001
Erythema/swelling	32 (4.1%)	58 (10.9%)	2.63 (1.73–3.99)	<0.001
Bleeding/exudate	21 (2.7%)	31 (5.8%)	2.14 (1.25–3.69)	0.005
Subcutaneous hematoma	2 (0.3%)	3 (0.6%)	2.18 (0.37–12.99)	0.403
Infection	1 (0.1%)	0 (0.0%)	Not estimable	1.000

RR, risk ratio; CI, confidence interval. Component events were summarized separately according to closure method and could overlap within the same patient. Crude RRs and 95% CIs were calculated when estimable.

*P* values were calculated using the Pearson chi-square test or Fisher exact test, as appropriate.

**Figure 3:**
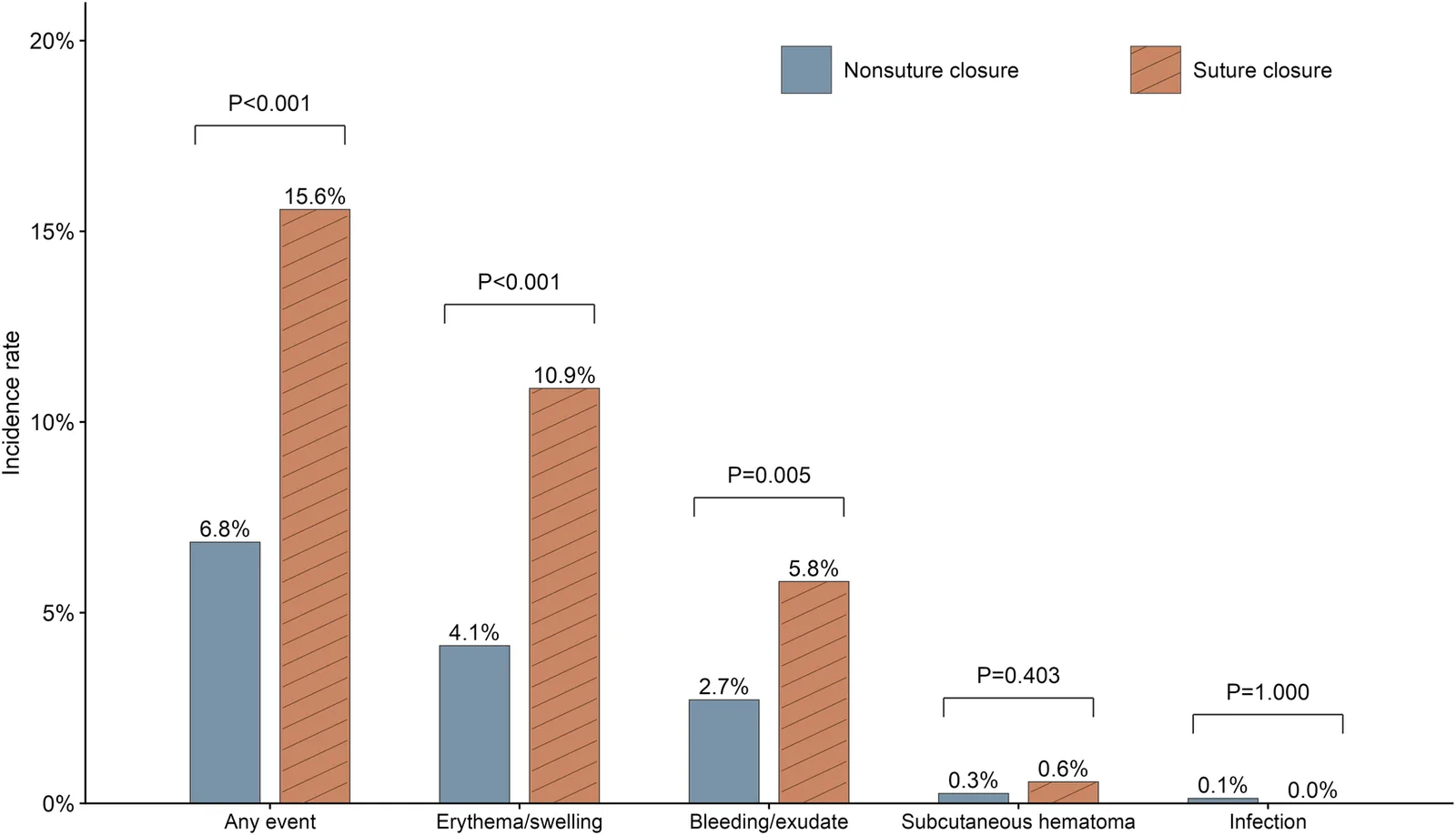
Incidence of early puncture-site wound event types by closure method.

## 4 Discussion

In this single-center cohort of patients with OVCFs who underwent PVP, 10.4% had recorded early puncture-site wound events within 2 weeks after surgery. Factors independently associated with the outcome after adjustment were suture closure, diabetes, elevated INR, and multilevel treatment, whereas age, sex, and BMI were not independently associated with the composite outcome.

Using an early wound composite outcome is consistent with the distribution of events after vertebral augmentation. Large-scale data indicate that SSI after vertebroplasty or kyphoplasty is uncommon. In a Korean nationwide database study including 23,868 vertebroplasty or kyphoplasty procedures, SSI occurred in 47 cases, with an incidence of 0.20% (6). Studies focusing on deep infection after vertebral augmentation similarly show that infection can have serious consequences but occurs infrequently (7, 18–20). By contrast, early puncture-site wound events, such as erythema or swelling, exudate, and small hematoma, are more common and more directly affect early wound observation, post-discharge communication, and decisions about whether additional wound management is needed. These events should be interpreted as early local wound findings rather than equivalent wound-healing failure, because most component events were mild local findings and severe infection was rare. However, previous PVP studies have rarely treated early puncture-site wound events as the analytical focus. Most have concentrated on severe complications, including cement leakage, neurologic events, pulmonary cement embolism, refracture, and deep spinal infection, while wound closure method is usually embedded in procedural descriptions and is not evaluated together with patient status and treatment extent (5, 13–17, 21–23). By modeling early puncture-site wound events as an outcome, this study provides new evidence for a problem that may influence postoperative management but has been underemphasized in previous research.

The association between diabetes and early puncture-site wound events is consistent with broader surgical evidence. A systematic review and meta-analysis across multiple surgical specialties showed that diabetes was independently associated with SSI (11). A recent meta-analysis of orthopedic SSI also identified diabetes as a significant patient-related risk factor (10). Zheng et al. (24) likewise reported an association between diabetes and tuberculous or nontuberculous spondylitis after PVP or kyphoplasty. Although the outcome in the present study was earlier and more superficial than these infectious outcomes, the direction of association is biologically plausible. Diabetes can delay wound repair through impaired microvascular perfusion, insufficient local angiogenesis, prolonged inflammation, reduced immune cell function, and impaired fibroblast and keratinocyte responses (25, 26). Although glycemic control, microvascular perfusion, and inflammatory pathways were not directly measured in this study, these previously described mechanisms provide a biologically plausible explanation for the observed association.

Elevated INR was associated with the composite early puncture-site wound outcome. The Cardiovascular and Interventional Radiological Society of Europe clinical practice manual recommends attention to anticoagulation intensity and INR level for spinal procedures, and states that patients receiving warfarin should have INR confirmed to be within the normal range before the procedure (27). Marked coagulation abnormalities are usually considered a reason to postpone elective PVP. Therefore, the focus of this study was not patients with severe coagulation disorders, but the risk difference between patients with normal INR and those with mildly elevated INR within a clinically acceptable range. Modeling INR as >1.2 used a clinically recognizable threshold and avoided imposing a linear assumption when the perioperative INR range was narrow. Although coagulation reserve was not directly measured and bleeding-specific associations were not analyzed, previous reports of bleeding complications and hidden blood loss provide biological context for the observed association (12, 28).

Previous studies indicate that multilevel PVP can generally be performed safely. Tan et al. reported the feasibility of multilevel PVP and described postoperative technical or radiographic events such as cement leakage, whereas Kapetanakis et al. evaluated multilevel revision PVP performed for symptomatic refracture, cement failure, or additional symptomatic levels after previous procedures and reported no new perioperative complications in a small cohort (29, 30). These studies mainly evaluated pain relief, mobility, and major technical complications rather than superficial puncture-site wound events. Thus, although they support the feasibility of multilevel PVP, they do not exclude a possible association between greater treatment extent and increased wound events. In the present study, the composite outcome was counted once per patient rather than per puncture site. Multilevel PVP may involve more puncture routes, repeated soft-tissue channels, and a longer procedure. Even when each individual puncture site is small, the cumulative puncture exposure associated with multilevel treatment may increase the opportunity for exudate, inflammatory reaction, or small hematoma. The adjusted RR for multilevel treatment was 1.73, suggesting that treatment extent remained associated with early wound events after adjustment for suture closure, diabetes, elevated INR, age, sex, and BMI. Previous spine surgery-related studies support this direction, as an increased number of treated levels has been associated with a higher risk of postoperative adverse outcomes (12, 24, 31, 32).

Suture closure showed a consistent association with early puncture-site wound events in both crude and adjusted analyses. The incidence of any early event was higher in patients with suture closure than in those with nonsuture closure (15.6% vs. 6.8%), and erythema or swelling and exudate showed the same direction. After adjustment, suture closure remained associated with the composite outcome (adjusted RR: 2.24). To reduce the possibility that this association merely reflected wounds that required suturing because they were more difficult, the cohort excluded patients with uncontrolled coagulation disorders, concomitant open spinal surgery, intraoperative puncture-site incision, malignant tumor or abnormal immune status, and missing key data. Although these restrictions cannot fully eliminate unrecorded selection bias related to local tissue condition or surgeon judgment, they reduce the likelihood that the comparison was driven mainly by wounds that required remedial suturing. In a study of percutaneous vertebral body stenting, Yang et al. (8) found that persistent intraoperative bleeding was the main reason for conversion from skin adhesive tape closure to suture closure. Unlike that vertebral body stenting study, our study examined elective PVP, used a 2-week patient-level composite of puncture-site wound events, and adjusted for patient factors, coagulation status, and treatment extent. Schlager et al. (9) and Liu et al. (10) found that suture or local wound management could be related to skin irritation, infection, blood-stained dressings, or other early wound problems. These studies suggest that puncture wounds after percutaneous vertebral augmentation can often heal without routine suturing, whereas suturing may be associated with contexts involving wound erythema, bleeding, or other local issues. In a larger PVP cohort, the present study further showed that suture closure was associated with a higher incidence of early wound events, especially erythema or swelling and exudate.

A meta-analysis including 45 studies and 1,572,160 patients suggested that higher BMI, low albumin, higher ASA class, longer operative time, and repeated surgery were associated with poor wound healing (10). In the present study, age and BMI did not reach statistical significance in the primary model, but both adjusted RRs were greater than 1. Therefore, the overall direction was not inconsistent with previous evidence. The lack of statistical significance may be related to the relatively mild severity of the endpoint events and the limited number of events in this study.

This study has several limitations. The retrospective single-center design may still be subject to residual confounding, including the influence of unrecorded intraoperative conditions on wound management. Puncture-site wound events were identified from inpatient records, postoperative dressing-change records, wound-clinic records, and documented telephone follow-up information; very mild erythema or transient bleeding may have been missed, recording thresholds may not have been fully consistent, and the intensity of wound assessment could not be standardized or quantified. In addition, postoperative contact with healthcare personnel may have differed between patients with and without sutures, particularly when suture removal or wound-clinic review was required, which may have influenced the detection and recording of mild wound events. The composite outcome improved event capture but combined events with different mechanisms and severity. The original records did not consistently capture event duration, additional care, revisits, hospitalization, or patient discomfort, so the management burden of these events could not be quantified. The number of infections within the 2-week follow-up window was very small, preventing stable modeling of SSI alone or assessment of later infections. The findings mainly apply to patients with OVCFs undergoing elective PVP under similar perioperative diagnosis and treatment pathways, and should not be directly generalized to open spinal surgery, patients with malignant tumors or immunosuppression, or puncture sites requiring remedial closure.

## 5 Conclusion

In this single-center retrospective cohort of patients with OVCFs who underwent elective PVP, 10.4% had recorded early puncture-site wound events within 2 weeks after surgery. Diabetes, elevated INR, multilevel treatment, and suture closure were associated with higher adjusted risk, whereas age, sex, and BMI were not independently associated with the outcome in the primary model. These results suggest that early wound status after PVP is associated with metabolic and coagulation status, treatment extent, and local puncture-site management. These findings may help identify priorities for postoperative wound observation after elective PVP.

## Data Availability

The datasets presented in this article are not readily available because the dataset contains de-identified clinical information derived from hospital medical records. Access is restricted to protect patient privacy and is available from the corresponding author upon reasonable request, subject to approval by the institution and compliance with applicable ethical and data-protection requirements. Requests to access the datasets should be directed to libei@fjmu.edu.cn.
